# Responding Rapidly to FDA Drug Withdrawals: Design and Application of a New Approach for a Consumer Health Website

**DOI:** 10.2196/jmir.8.3.e16

**Published:** 2006-09-06

**Authors:** Peter J Embi, Prasad Acharya, Mark McCuistion, Charles P Kishman, Doris Haag, Stephen Marine

**Affiliations:** ^1^University of Cincinnati Medical CenterCincinnatiOHUSA; ^2^Boonshoft School of MedicineWright State UniversityDaytonOHUSA

**Keywords:** Consumer health information, drug and narcotic control, cyclooxygenase inhibitors, adverse effects, time factors

## Abstract

**Background:**

Information about drug withdrawals may not reach patients in a timely manner, and this could result in adverse events. Increasingly, the public turns to consumer health websites for health information, but such sites may not update their content for days or weeks following important events like Food and Drug Administration (FDA) drug withdrawal actions. There is no recognized standard for how quickly consumer health websites should respond to such events, and reports addressing this issue are lacking.

**Objective:**

The objective of this study was to develop and implement an approach to enhance the efficiency with which a consumer health website (NetWellness.org) responds to FDA drug withdrawal actions.

**Methods:**

Evaluation of the current approach used by NetWellness staff to update content affected by FDA action revealed a slow process driven by the goal of performing thorough and comprehensive review and editing. To achieve our desired goal of accurately updating affected content within 24 hours of FDA action, we developed a strategy that included rapid updating of affected Web pages with warning boxes and hyperlinks to the information about the withdrawal. With the next FDA withdrawal event, that of valdecoxib (Bextra) on April 7, 2005, we applied this new approach, observed the time and resource requirements, and monitored the rate at which consumers viewed the updated information to gauge its potential impact.

**Results:**

Application of the new approach allowed one person to modify the affected Web pages in less than 1 hour and within 18 hours of the FDA announcement. Using the old strategy, response to a similar event, the withdrawal of rofecoxib (Vioxx) 6 months earlier, had taken over 3 weeks and the efforts of several personnel. Updated valdecoxib content received 188 hits within the first month and 4285 hits within 1 year.

**Conclusions:**

Rapid updating of a consumer health website's content in response to an FDA drug withdrawal event was easily accomplished by applying the approach described. This allowed consumers to view accurate information regarding the withdrawn drug much sooner than would otherwise have been the case. Given that consumers increasingly turn to websites for their health information, adoption of a rapid response standard for important health events like FDA drug withdrawals should be considered by the consumer health informatics community.

## Introduction

Despite the extensive evaluation process before a drug is approved for use by the US Food and Drug Administration (FDA), some drugs are occasionally found to have unanticipated significant adverse effects after their release. In such cases, drugs may be withdrawn from the market after having been in wide use, sometimes for years.

A high profile example involved rofecoxib (trade name Vioxx), which was withdrawn on September 30, 2004 [1]. This nonsteroidal anti-inflammatory drug (NSAID) is a member of the popular coxib class of drugs (selective cyclooxygenase-2 [COX-2] inhibitors). Post-marketing research data indicated that rofecoxib had unanticipated health effects, which led to its withdrawal from the market. At the time of its withdrawal, rofecoxib was in use by some 2 million people in the United States alone, making this the largest prescription drug withdrawal in US history [2].

Informing the public about drug withdrawals like this one often involves a combination of efforts by the FDA and the drug’s manufacturer as well as pharmacy-level withdrawal of the drug to prevent further sales [1,3-5]. However, the timeliness and manner of informing health care personnel and patients of drug withdrawals varies [6-8]. There is evidence that patients continue to use medications for some time after withdrawal from the market, occasionally with adverse consequences [9,10]. Indeed, even brief prolongation of the use of drugs like rofecoxib may be detrimental to some users, further emphasizing the importance of informing the public of drug withdrawals as quickly as possible [11].

The World Wide Web (Web) is a major source of health information for millions of consumers [12,13]. While the quality of sites vary, some trusted resources provide credible evidence and serve as an important source of health information for many consumers [14]. One such trusted website is NetWellness (www.netwellness.org), a non-profit, consumer health information portal with ask-an-expert service. NetWellness provides anonymous access to all content, including over 29000 consumer-submitted questions and expert responses authored by over 380 volunteer health sciences faculty experts from NetWellness’ three Ohio medical research university partners—University of Cincinnati, Case Western Reserve University, and The Ohio State University [15,16]. This content includes thousands of instances of drug-specific information. While it is the procedure of NetWellness to review and update content on at least an annual basis, events such as FDA drug withdrawals trigger more frequent updates. However, as described in greater detail below, the baseline process for performing such updates is often inefficient.

Given the public’s growing use and reliance on such websites for their health information and the importance of rapidly informing consumers about significant drug events, the purpose of this project was to devise and implement an improved process that would allow for efficient and consistently effective updating of content in response to drug withdrawals. Our goal was to update all instances of the withdrawn drug name (generic and trade) in NetWellness within 24 hours of FDA action so that visitors would have the benefit of full and accurate health information.

## Methods

### Baseline Approach to Drug Information Updates on NetWellness

An example of a drug update resulted from the withdrawal of rofecoxib from the world market on September 30, 2004. Interviews with NetWellness personnel revealed the following process. Upon learning of this withdrawal through media reports, NetWellness personnel performed a manual search of the NetWellness database using built-in search engines to identify Web pages containing the terms “rofecoxib” or “Vioxx.” Once identified, Web pages containing those terms were archived, and authors were asked to evaluate the content and assess whether the page should be altered (and if so, how) or permanently archived. Finally, changes to the pages were made and revised pages were re-posted. The entire process took about 3 weeks to complete, owing mostly to delays in author responsiveness.

While the updating process was ultimately effective, it was quite time-consuming. As a result, information about the drug’s withdrawal remained unavailable on NetWellness for weeks after it was withdrawn from the market. Furthermore, the process relied on the vigilance of NetWellness personnel to monitor relevant information sources, such as news items, in order to discover that such FDA actions had occurred, leaving open the possibility that such a process may not even be initiated for some time after an FDA action.

### New Approach to Drug Information Updates on NetWellness

The first step toward achieving our objective was to determine a way to consistently and efficiently become aware of all instances of FDA drug withdrawal actions. Previously, NetWellness personnel learned about drug withdrawals through periodic review of reports in medical or popular media. For the new process, we considered several alternatives and opted to use the FDA’s MedWatch E-list safety alert email reporting service. Key NetWellness personnel registered to receive and monitor the email alerts on a daily basis.

Next, we devised a new simplified method for updating the NetWellness database. As with the previous process, NetWellness personnel would query the database for all instances of the drug name (trade and generic). All instances identified would then be replaced with hyperlinked text using an automated find-and-replace function built into the NetWellness content management system. Updated pages would also contain a warning box at the foot of the page indicating the availability of important new information about the drug in question. Hyperlinked text items in the main body of the page or the footer would point to a new NetWellness page containing a warning about the FDA withdrawal and providing additional links to the FDA or drug manufacturer website for more information. Development and lab tests of this approach were completed in February 2005.

## Results

On April 7, 2005, citing health concerns, the FDA issued an announcement that sales of another coxib medication, valdecoxib (trade name Bextra), were to cease immediately [17]. Designated NetWellness personnel received the notice via email from the MedWatch mailing list and immediately activated the new updating procedure described above. All NetWellness Web pages mentioning the drug were updated with links to a newly created valdecoxib warning page. An example of a Web page before and after modification is shown in Figure 1. The valdecoxib warning page describing the withdrawal and providing a link to the relevant FDA Web page is shown in Figure 2.

**Figure 1a:**
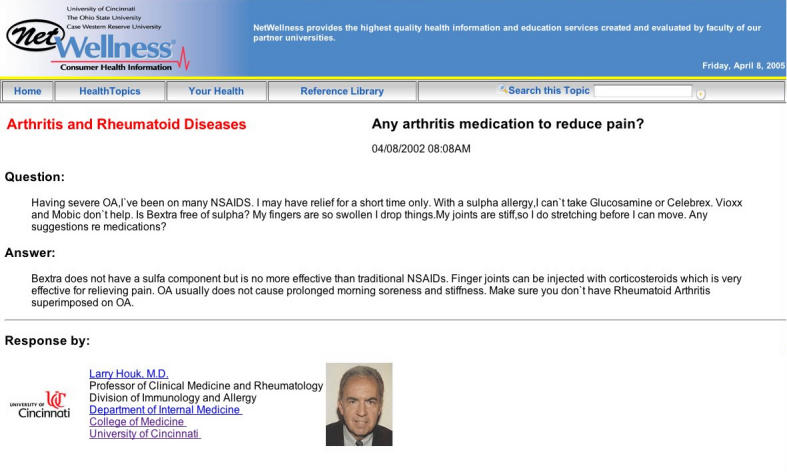
Example of a NetWellness content page before and after updates to reflect FDA withdrawal of valdecoxib (Bextra). (a) Before update

**Figure 1b:**
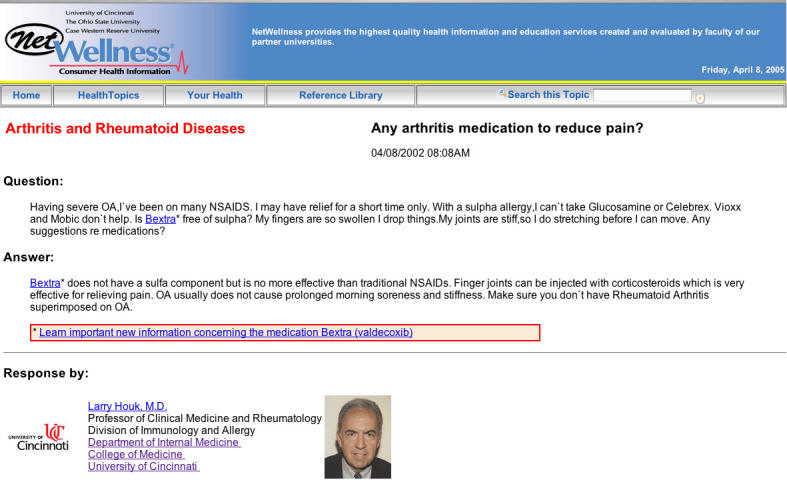
(b) After update

**Figure 2:**
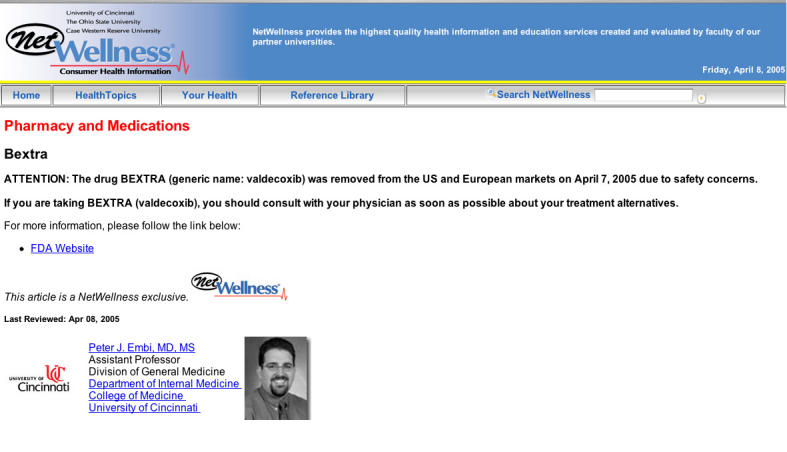
Drug withdrawal information page

By following the procedure outlined above, updates to the system were completed within 18 hours of the FDA drug recall announcement. This represented a significant improvement over the weeks it had taken to fully respond to the previous FDA drug recall of rofecoxib six months earlier. Moreover, updates to the content were accomplished with a fraction of the resources previously required.

In the first month following the content update, NetWellness valdecoxib-related Web pages received 188 hits, with 156 (83%) following the links to the new warning page. As of April 8, 2006, 1 year after the withdrawal of valdecoxib, updated Web pages had received 4285 hits, with 1017 (24%) of those viewing the warning information page.

## Discussion

Given the public’s growing use of the Web for health information, it is important that Web-based consumer health content is kept up-to-date, particularly important content like that regarding withdrawal of a potentially harmful medication [18]. Unfortunately, many online health information providers do not respond rapidly enough to such important events, with most taking several days or longer to respond to the valdecoxib recall [19]. In the case of NetWellness, a paradigm shift was necessary in order to meet the challenge of a more rapid response time. The previous system of thorough review and revision of affected content throughout the website was replaced with a process that concentrated instead on rapid updating of affected content in a time- and resource-efficient manner.

Following our test event with valdecoxib, we observed many consumers reviewing the new health information and potentially benefiting from the rapid response. As might be expected, the proportion of those viewing the warning information was highest during the initial month after recall, although a substantial number of users also reviewed content many months later. This suggests that consumers’ information needs regarding withdrawn drugs may persist long after the event, and it seems to support the importance of updating rather than simply permanently archiving such content.

This study has some limitations. The anonymous design of our website did not allow formal evaluation of the impact of the new update approach on NetWellness users, and we do not know to what extent users had already learned of the FDA action when they reviewed the updated content.

Future steps include evaluating this approach more rigorously and developing processes for responding to the far more frequent, if less extreme, drug warnings announced by the FDA. In order to respond even quicker in the future, we are working to automate as much of the updating process as possible. While human review is currently the best way to determine the significance of an FDA announcement and initiate an appropriate response, advancements in the structure and format of FDA-generated electronic information feeds may soon facilitate a fully automated response.

This paper demonstrates that a simple approach can allow for rapid updating of critical content on consumer health websites in response to a drug’s withdrawal from the market. Given the importance of quickly performing such updates and the feasibility of doing so, we recommend that the consumer health informatics community adopt a 24-hour response standard for updating affected website content following a drug withdrawal event.

## Abbreviations

COX-2: cyclooxygenase-2
FDA: Food and Drug Administration
NSAID: nonsteroidal anti-inflammatory drug
